# Predictability of polygenic risk score for progression to dementia and its interaction with *APOE* ε4 in mild cognitive impairment

**DOI:** 10.1186/s40035-021-00259-w

**Published:** 2021-08-31

**Authors:** Jung-Min Pyun, Young Ho Park, Keon-Joo Lee, SangYun Kim, Andrew J. Saykin, Kwangsik Nho, for the Alzheimer’s Disease Neuroimaging Initiative

**Affiliations:** 1 Department of Neurology, Uijeongbu Eulji Medical Center, Eulji University, Uijeongbu, Republic of Korea; 2 Department of Neurology, Seoul National University Bundang Hospital and Seoul National University College of Medicine, Seongnam, Republic of Korea; 3 Department of Radiology and Imaging Sciences, and the Indiana Alzheimer Disease Center, Indiana University School of Medicine, Indianapolis, IN USA; 4 Department of Medical and Molecular Genetics, Indiana University School of Medicine, Indianapolis, IN USA; 5 Center for Computational Biology and Bioinformatics, Indiana University School of Medicine, Indianapolis, IN USA

**Keywords:** Polygenic risk score, Mild cognitive impairment, Alzheimer’s disease, Disease progression, *APOE* ε4

## Abstract

**Background:**

The combinatorial effect of multiple genetic factors calculated as a polygenic risk score (PRS) has been studied to predict disease progression to Alzheimer’s disease (AD) from mild cognitive impairment (MCI). Previous studies have investigated the performance of PRS in the prediction of disease progression to AD by including and excluding single nucleotide polymorphisms within the region surrounding the *APOE* gene. These studies may have missed the *APOE* genotype-specific predictability of PRS for disease progression to AD.

**Methods:**

We analyzed 732 MCI from the Alzheimer’s Disease Neuroimaging Initiative cohort, including those who progressed to AD within 5 years post-baseline (*n* = 270) and remained stable as MCI (*n* = 462). The predictability of PRS including and excluding the *APOE* region (PRS_+*APOE*_ and PRS_−*APOE*_) on the conversion to AD and its interaction with the *APOE* ε4 carrier status were assessed using Cox regression analyses.

**Results:**

PRS_+*APOE*_ (hazard ratio [HR] 1.468, 95% CI 1.335–1.615) and PRS_−*APOE*_ (HR 1.293, 95% CI 1.157–1.445) were both associated with a significantly increased risk of MCI progression to dementia. The interaction between PRS_+*APOE*_ and *APOE* ε4 carrier status was significant with a *P*-value of 0.0378. The association of PRSs with the progression risk was stronger in *APOE* ε4 non-carriers (PRS_+*APOE*_: HR 1.710, 95% CI 1.244–2.351; PRS_−*APOE*_: HR 1.429, 95% CI 1.182–1.728) than in *APOE* ε4 carriers (PRS_+*APOE*_: HR 1.167, 95% CI 1.005–1.355; PRS_−*APOE*_: HR 1.172, 95% CI 1.020–1.346).

**Conclusions:**

PRS could predict the conversion of MCI to dementia with a stronger association in *APOE* ε4 non-carriers than *APOE* ε4 carriers. This indicates PRS as a potential genetic predictor particularly for MCI with no *APOE* ε4 alleles.

**Supplementary Information:**

The online version contains supplementary material available at 10.1186/s40035-021-00259-w.

## Background

Predicting disease progression to Alzheimer’s disease (AD) from mild cognitive impairment (MCI) is critical for identifying individuals for opportune intervention in clinical management and for optimization of target participants in clinical trials. The use of individualized genetic profile is thriving with a pursuit of precision medicine and is becoming approachable with shared large data, which may facilitate customized risk prediction of disease development and progression. The heritability of late-onset AD (LOAD) is high, accounting for 60%–80% [1], and genome-wide association studies (GWAS) have identified multiple genetic factors associated with AD including the well-known *APOE* ε4 allele [2, 3]. The combinatorial effect of these multiple genetic factors can be calculated as a polygenic risk score (PRS). PRS has been widely evaluated in AD research regarding its value in AD risk prediction, its relations with conventional biomarkers of AD, and its prediction of disease conversion from MCI to AD [4]. In particular, the predictability of PRS on disease progression from MCI to AD is substantially affected by the presence of *APOE* ε4 allele, and PRS excluding the *APOE* region may or may not predict disease progression [5–8].

In this study, we aimed to investigate the predictability of PRSs including and excluding single nucleotide polymorphisms (SNPs) within the region surrounding the *APOE* gene, on MCI progression to AD, and the interaction between PRS and *APOE* ε4 alleles. In addition, the predictability of hippocampal volume on MRI and amyloid PET were assessed, which are well-known predictors of disease progression. Furthermore, we identified SNPs used for PRS calculation and then performed enrichment analysis to explore the implicated biological pathways.

## Methods

### Participants

Data used in this study were obtained from the Alzheimer’s Disease Neuroimaging Initiative (ADNI) database (http://adni.loni.usc.edu/). ADNI was launched in 2003 as a public–private partnership. The primary goal of ADNI has been to test whether serial MRI, PET, other biological markers, and clinical neuropsychological assessment can be combined to measure the progression of MCI and early AD [9]. In this study, patients with a diagnosis of MCI and with available GWAS data, older than 60 years at baseline assessment, and having at least one or more follow-up visits, were included. We focused on the genetic risk estimation of LOAD, in which the threshold cutoff of 60 years is commonly used. The primary outcome of this study was MCI conversion to dementia during a follow-up period up to 5 years post-baseline. MCI was diagnosed when there was objective memory impairment but without meeting the criteria for dementia [9]. The MCI participants had Mini-Mental State Examination (MMSE) scores between 24 and 30, memory performance scores approximately 1 standard deviation below expected education-adjusted norms, and a clinical dementia rating score of 0.5.

### Genotyping and imputation

Genotyping for ADNI was performed using blood DNA samples and a combination of Illumina GWAS array platforms (Illumina Human610-Quad BeadChip, Illumina HumanOmni Express BeadChip, and Illumina HumanOmni 2.5 M BeadChip) [10]. *APOE* genotyping was separately conducted using previously described standard methods to yield the *APOE* ε4 allele-defining SNPs (rs429358, rs7412) [10, 11]. Using PLINK 1.9 (www.cog-genomics.org/plink2/) [12], we performed standard quality control (QC) procedures for samples and SNPs as described previously [13]: (1) for SNPs: SNP call rate < 95%, Hardy–Weinberg *P* value < 1 × 10^–6^, and minor allele frequency (MAF) < 1%; (2) for samples: sex inconsistencies, and sample call rate < 95%. Then, to prevent spurious associations due to population stratification, we used multidimensional scaling analysis to select only non-Hispanic participants of European ancestry that clustered with HapMap CEU (Utah residents with Northern and Western European ancestry from the CEPH collection) or TSI (Toscani in Italia) populations (Additional file 1: Fig. S1) [14, 15]. After QC procedures, we imputed un-genotyped SNPs separately in each platform using MaCH with the Haplotype Reference Consortium data as a reference panel [16, 17]. Following the imputation, we imposed an *r*^2^ value of 0.30 as the threshold to accept the imputed genotypes [18].

### Imaging biomarkers

T1-weighted brain MRI scan was processed with FreeSurfer version 5.1 to measure hippocampal and intracranial volumes [19]. For assessment of cortical amyloid accumulation, we used preprocessed (coregistered, averaged, standardized image and voxel size, uniform resolution) [^18^F] florbetapir PET scans [20] and calculated the mean standardized uptake value ratio (SUVR) using a whole cerebellum reference region as previously described [21]. In our analysis, amyloid burden on PET was dichotomized as positive when SUVR ≧ 1.17 and negative when SUVR < 1.17 [22].

### Calculation of PRS

PRSs were calculated using the software PRSice v2.3.1.e [23]. The GWAS summary statistics from Jansen et al. were used as a base dataset [2] and the phase 3 genetic data from the 1000 Genomes Project [24] for non-Hispanic participants of European ancestry were used to calculate the linkage disequilibrium structure. To investigate the optimal *P*-value threshold to select AD-associated SNPs, we iterated *P*-value thresholds of 1 × 10^–6^, 1 × 10^–5^, 1 × 10^–4^, 1 × 10^–3^, 1 × 10^–2^, 0.05, and 0.5. PRSs were separately calculated including and excluding SNPs within the 1 Mb-region surrounding the *APOE* gene, which was defined as a region from 1 Mb before rs429358 to 1 Mb after rs7412. The numbers of SNPs used for PRS calculation depending on the various *P*-value thresholds are shown in Additional file 1: Table S1. In addition, PRS was z-transformed based on the PRS distribution among amyloid PET-negative cognitively normal participants (*n* = 138).

### Statistical analysis

All statistical analyses were made using the R v4.0.2 software (www.R-project.org). Comparisons of demographics between stable MCI, who remained stable at MCI during 5 years after baseline, and progressive MCI, who progressed to dementia within 5 years, were made with Mann–Whitney U test and Chi-squared test as appropriate.

To assess the predictability of PRS, hazard ratio (HR) of PRS in z-scores was obtained with Cox regression analysis. PRSs including and excluding SNPs within the 1 Mb-region surrounding the *APOE* gene were analyzed and represented as PRS_+*APOE*_ and PRS_−*APOE*_, respectively. PRS analyses were performed in all MCI patients, MCI carrying the *APOE ε*4 alleles, and MCI not carrying the *APOE ε*4 alleles, separately. Cox regression analyses of PRS are presented as Kaplan Meier curves. The interaction between PRSs and *APOE ε*4 carrier status was also assessed. To evaluate the predictability of MRI hippocampal volume, the hippocampal volume was divided by the intracranial volume and z-transformed based on the distribution of values within amyloid PET-negative cognitively normal older adults. The HR of *APOE ε*4 carriers was compared to *APOE ε*4 non-carriers, and the HR of amyloid PET-positive MCI was compared to amyloid PET-negative MCI. All analyses were adjusted with age and sex, and additionally with MRI field strength in hippocampal volume analysis. Statistical significance was set at *P* < 0.05.

### Functional interpretation of SNPs used for PRS calculation

Functional mapping and annotation of SNPs used for PRS calculation was performed with the FUMA v1.3.6a software [25]. The GWAS summary statistics of SNPs were obtained from Jansen et al. [2]. All known SNPs that had *r*^2^ ≥ 0.6 with one of the independent significant SNPs used for PRS calculation, were also included for gene mapping using FUMA. Gene mapping was conducted by three methods, including positional mapping, expression quantitative trait loci (eQTL) mapping, and chromatin interaction mapping. In the positional mapping, SNPs were mapped to genes with a maximum distance of 10 kb. eQTL data from the eQTL catalogue (BrainSeq brain) [26], the Blood eQTLs (Westra et al. (2013) Blood eQTL Browser [27], Zhernakiva et al. (2017) BIOS QTL Browser [28]), the BRAINEAC (averaged expression of 10 brain regions including frontal cortex, hippocampus, occipital cortex, temporal cortex, cerebellar cortex, inferior olivary nucleus, putamen, substantia nigra, thalamus, and intralobular white matter) [29], and the GTEx v8 Brain (cortex, frontal cortex BA9, hippocampus)[30] datasets were used for eQTL mapping, and only significant SNP-gene pairs with a false discovery rate (FDR) cutoff of 0.05 were used. The chromatin interaction mapping was performed with data from the HiC (GSE87112) dorsolateral prefrontal cortex and hippocampus [31] with FDR cutoff of 1 × 10^–6^ and promotor region window of 250 bp up- and 500 bp down-stream of the transcription start site. The possible biological processes (BP), molecular functions (MF), and involved cellular components (CC) of mapped genes were explored by gene set enrichment analysis using data from the gene ontology (GO) [32, 33]. Statistical significance was set at adjusted *P*-value < 0.05.

## Results

A total of 907 MCI participants from the ANDI cohort were assessed for study eligibility. After excluding 48 participants younger than 60 years, 68 without follow-up visit, and 59 without available GWAS data, 732 participants were finally included for the analysis. Of the 732 MCI participants, 270 MCI patients (36.8%) were converted to dementia within 5 years (“progressive MCI”) and 462 MCI patients remained at MCI (“stable MCI”). There was no significant difference in age, sex, or education level at baseline between the two groups. The progressive MCI group had a significantly higher proportion of *APOE ε*4 allele-carriers, lower MMSE scores and higher clinical dementia rating sum of boxes (CDR-SB) scores at baseline (Table 1).

**Table 1:** Baseline demographics of  participants

	Stable MCI (*n* = 462)	Progressive MCI (*n* = 270)	*P*-value
Age, years	73 (67–79)	74 (69–79)	0.109
Female, *n* (%)	179 (38.74)	105 (38.89)	1.000
Education, years	16 (14–18)	16 (14–18)	0.481
*APOE ε*4 carrier, *n* (%)^*^	196 (42.52)	182 (67.41)	< 0.001
MMSE	28 (27–29)	27 (26–28)	< 0.001
CDR-SB	1 (0.5–1.5)	2 (1.0–2.5)	< 0.001

Data are presented as median (interquartile range) unless otherwise specified

^*^Data unavailable for 1 subject

We examined seven *P*-value thresholds to find the optimal threshold for selection of AD-associated SNPs, and the model with threshold* P* < 1 × 10^–5^ showed the best predictability and was used for further analysis (Additional file 1: Table S2).

**Fig. 1:**
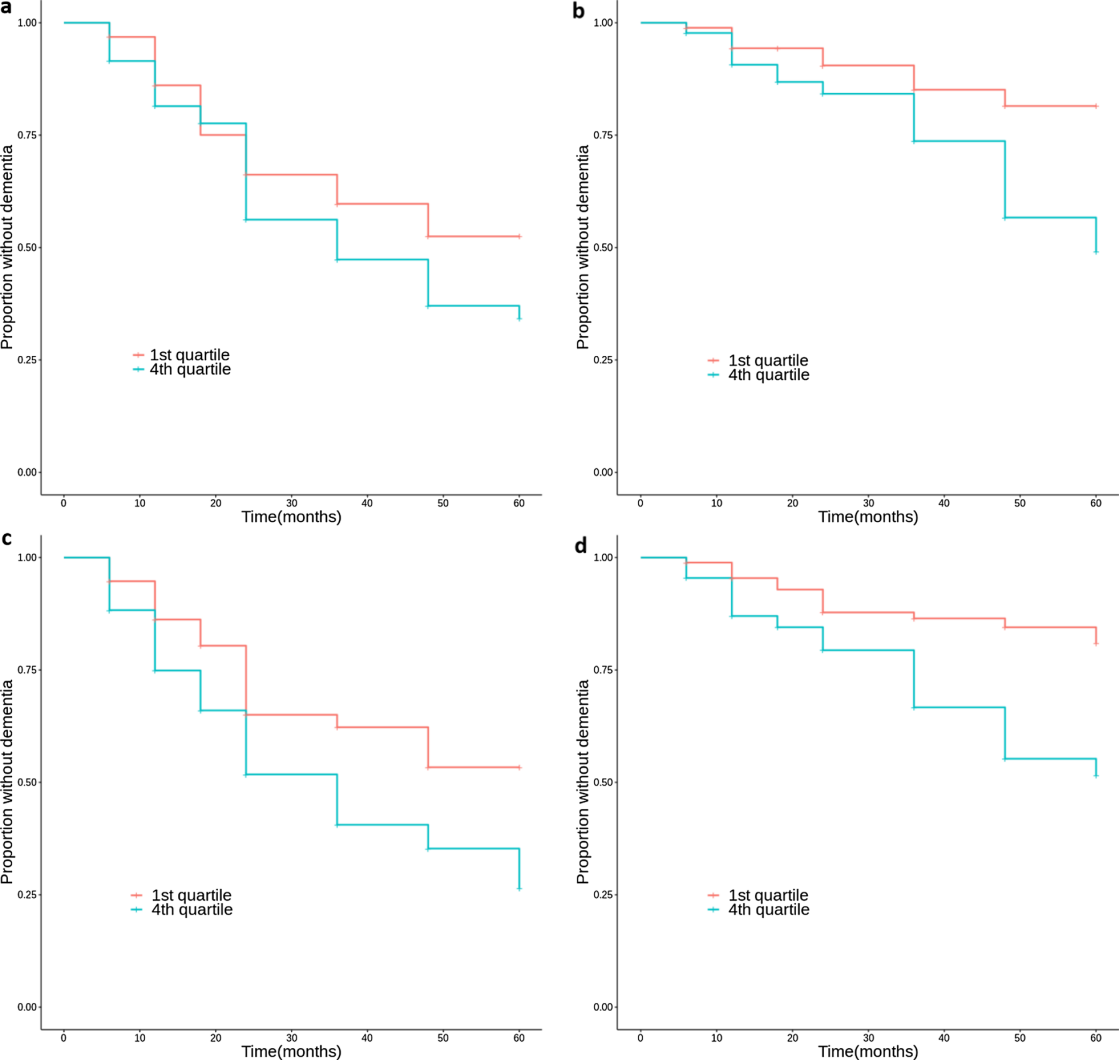
Kaplan Meier curves for disease progression from mild cognitive impairment to dementia of the lowest PRS group (1st quartile) and the highest PRS group (4th quartile). PRS_+*APOE*_ in MCI with (**a**) and without *APOE* ε4 (**b**). PRS_−*APOE*_ in MCI with *APOE* ε4 (**c**) and without *APOE* ε4 (**d**) are presented

In the Cox regression analysis to assess the predictability for the conversion of MCI to AD, PRS_+*APOE*_ (HR 1.468, 95% CI 1.335–1.615) and PRS_−*APOE*_ (HR 1.293, 95% CI 1.157–1.445) both presented a significant association with the risk of disease progression (Table 2). PRS_+*APOE*_ showed significantly increased HRs in both MCI with and without *APOE* ε4 alleles. In particular, the risk association in MCI with no *APOE* ε4 alleles was stronger (HR 1.710, 95% CI 1.244–2.351) than MCI with *APOE* ε4 alleles (HR 1.167, 95% CI 1.005–1.355) (Table 2). The interaction between PRS_+*APOE*_ and *APOE* ε4 carrier status in Cox regression analysis was also significant, with a coefficient of − 0.372 (Table 3). PRS_−*APOE*_ also presented stronger risk association in MCI with no *APOE* ε4 alleles (HR 1.429, 95% CI 1.182–1.728) than in MCI with *APOE* ε4 alleles (HR 1.172, 95% CI 1.020–1.346). However, the interaction between PRS_−*APOE*_ and *APOE* ε4 carrier status was not significant, with a coefficient of − 0.174. HRs of PRS_+*APOE*_ and PRS_−*APOE*_ in MCI with and without *APOE* ε4 alleles are presented with Kaplan Meier curves (Fig. 1).

**Table 2:** Association of PRS, *APOE* ε4 status, hippocampal volume on MRI, and amyloid PET positivity with disease progression to dementia according to z-scores in all MCI participants, MCI with *APOE* ε4, and MCI without *APOE* ε4

	HR (95% CI), *P*-value		
	All MCI (*n* = 732)	MCI with *APOE* ε4 (*n* = 378)	MCI without *APOE* ε4 (*n* = 353)
PRS_+*APOE*_	1.468 (1.335–1.615) 2.30 × 10^–15^	1.167 (1.005–1.355) 4.16 × 10^–2^	1.710 (1.244–2.351) 9.30 × 10^–4^
PRS_−*APOE*_	1.293 (1.157–1.445) 5.19 × 10^–6^	1.172 (1.020–1.346) 2.47 × 10^–2^	1.429 (1.182–1.728) 2.19 × 10^–4^
*APOE* ε4 carrier status	2.678 (2.066–3.470) 9.70 × 10^–14†^	NA	NA
Hippocampal volume on MRI	0.563 (0.502–0.632) < 2.00 × 10^–16*^	0.538 (0.461–0.628) 3.55 × 10^–15†^	0.536 (0.435–0.660) 4.44 × 10^–9††^
Amyloid PET positivity	7.449 (4.199–13.215) 6.61 × 10^–12§^	5.011 (1.966–12.764) 7.31 × 10^–4∥^	6.500 (2.914–14.495) 4.77 × 10^–6¶^

PRS_+*APOE*_: PRS including SNPs within the 1 Mb-region surrounding the *APOE* gene; PRS_−*APOE*_: PRS excluding SNPs within the 1 Mb-region surrounding the *APOE* gene

Cox regression analysis of all variables were adjusted with age and sex, and additionally with MRI field strength in hippocampal volume analysis

Data unavailable for ^*^ 3 subjects, ^†^ 1 subject, ^††^ 2 subjects, ^§^ 343 subjects, ^∥^191 subjects, and ^¶^ 152 subjects

**Table 3:** Cox regression analysis of interaction between PRS and *APOE* ε4 carrier status in association with disease progression from MCI to AD

	Coefficient	Standard error	*P*-value
Age	0.034	0.009	1.94 × 10^–4^
Female	0.116	0.127	3.57 × 10^–1^
PRS_+*APOE*_	0.531	0.162	1.10 × 10^–3^
*APOE* ε4 carrier status	0.681	0.176	1.14 × 10^–4^
PRS_+*APOE*_ × *APOE* ε4 carrier status	− 0.372	0.179	3.78 × 10^–2^

PRS_+*APOE*_: PRS including SNPs within the 1 Mb-region surrounding the *APOE* gene

Next, we performed Cox regression analysis to assess the predictability of AD-related biomarkers such as amyloid PET positivity and hippocampal volume on MRI, separately, for the conversion of MCI to AD, using imaging biomarkers as independent variables. The MCI participants carrying the *APOE ε*4 allele showed increased HR (2.678, 95% CI 2.066–3.470) compared to the MCI not carrying the *APOE ε*4 allele. The hippocampal volume on MRI was related to a decreased risk of disease progression (HR 0.563, 95% CI 0.502–0.632), and the amyloid PET positivity predicted disease progression with HR of 7.449 (95% CI 4.199–13.215) (Table 2).

**Fig. 2:**
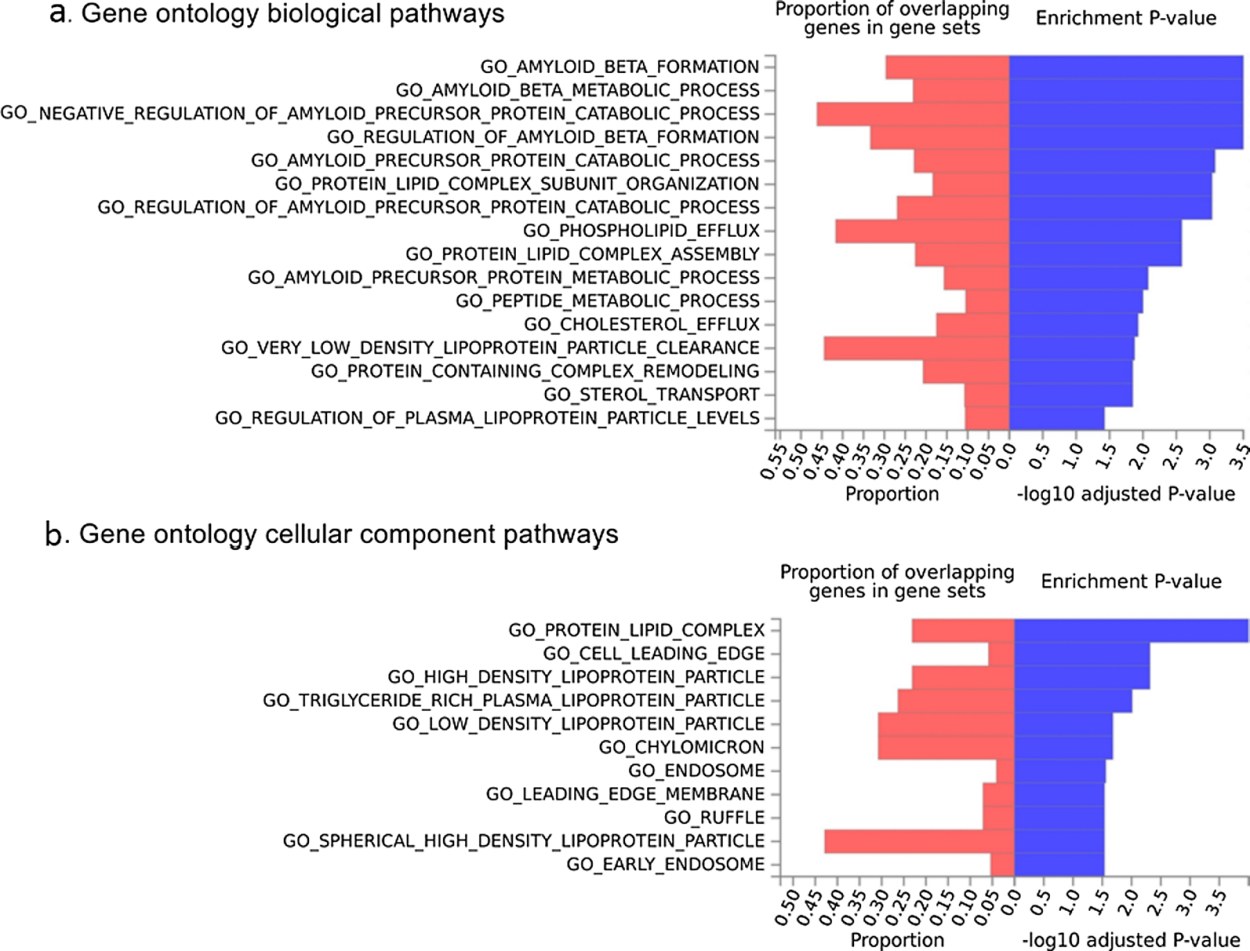
Gene ontology biological pathways and cellular component pathways

For gene mapping, all SNPs (*n* = 4967) that had *r*^2^ ≥ 0.6 with one of the independent SNPs (*r*^2^ < 0.05, *n* = 204) used to calculate PRS at a selection threshold of *P* < 1 × 10^–5^, were mapped on 424 genes. Among the 4967 SNPs, 3551 SNPs were used for positional mapping of 316 genes, 2898 SNPs for eQTL mapping of 264 genes, and 73 SNPs for chromatin interaction mapping of 19 genes. Among a total of 424 mapped genes, 133 genes were mapped by multiple independent significant SNPs. The mapped genes are listed in Additional file 2: Table S3. Gene set enrichment analysis yielded 16 significant GO BP pathways, 11 significant GO CC pathways, and no significant GO MF pathways (Fig. 2).

## Discussion

In this study, we demonstrated that PRS_+*APOE*_ was significantly associated with an increased risk of progression to dementia in MCI. PRS_−*APOE*_ was also related to an increased risk of progression, albeit less pronounced. Notably, the association of PRS with progression risk was stronger in *APOE* ε4 non-carriers than in *APOE* ε4 carriers in MCI, showing a significant interaction between PRS_+*APOE*_ and *APOE* ε4 carrier status on the conversion of MCI to dementia.

A previous study showed that the higher PRS, excluding chromosome 19 SNPs to avoid the *APOE* effect, could predict clinical progression to MCI/AD from non-demented status, with unstandardized β value of 0.49 in logistic regression analysis. PRS including chromosome 19 has also shown similar results with unstandardized β value of 0.43 [5]. In another study, while PRS with *APOE* is significantly associated with the progression risk from MCI to AD with HR of 1.59 (95% CI 1.31–1.78), PRS without *APOE* could not predict the progression with HR of 1.03 (95% CI 0.79–1.34) [6]. In the current study, we found comparable HRs for PRS with and without *APOE* and additionally confirmed different predictability between *APOE* ε4 carriers and non-carriers of the MCI participants.

Here, PRS_−*APOE*_ showed less prominent association with disease progression risk than PRS_+*APOE*_*,* which suggests that *APOE* is the strongest known risk gene for LOAD and affects the predictability of PRS. The *APOE* ε4 carriers show a  three- to four-fold increase of risk of AD development compared to the *APOE* ε4 non-carriers [34]. In addition to the effect of *APOE* alone, other AD-associated genes can interact with *APOE* to affect the disease course. A previous study has revealed that the SNP variants at *FYN* and *RNF219* loci are associated with decreased LOAD age-of-onset in *APOE* ε4 non-carriers but not in *APOE* ε4 carriers [35]. The *RNF219* variant is also related with the beta-amyloid (Aß) load in *APOE* ε4 non-carriers, but not in *APOE* ε4 carriers [35]. Alleles of the *CETP* gene could alter AD risk in an *APOE*-dependent manner [36]. In a meta-analysis, *CLU* is associated with AD only in *APOE* ε4 non-carriers and *PICALM* only in *APOE* ε4 carriers [37]. The SNPs used for PRS calculation in our study also included SNPs of *CLU* and *PICALM,* and the integrated effect of interaction between genes containing SNPs of PRS and *APOE* could manifest as a stronger progression risk in *APOE* ε4 non-carriers of our MCI cohort.

How the AD-associated genes impact AD pathology is not completely elusive, although the exact mechanism of the well-known *APOE* ε4 allele for amyloid clearance and aggregation and its association with AD-associated genes remain unclear [38]. Recent studies have found that the *APOE* ε4 allele is associated with Aß elevation and accumulation on PET, whereas PRS is more associated with faster cognitive decline in amyloid-positive status [39, 40]. This implies that *APOE* ε4 contributes to the initiation of amyloidopathy in an early stage, while other genetic variants are involved in disease progression. In addition, pathway analysis has shown that a large proportion of the AD-related pathways is associated with the *APOE* region. Furthermore, pathways such as the protein-lipid complex subunit organization and protein-lipid complex assembly have also been found to be involved in the AD risk independent of the *APOE* region [24, 39]. Again, these pathways were confirmed in our GO BP results. Another study investigating the contribution of five pathway-specific PRSs to AD risk has shown that the most involved pathway is Aß metabolism (29.6%) when the analysis included *APOE* variants, and immune response (45.5%) when the analysis excluded *APOE* variants [41].

However, the pathways related to *APOE* and PRS cannot be distinguished clearly, and a complex interaction of Aß, tau metabolism, cholesterol/lipid metabolism [42, 43], endosomal–lysosomal processing [44], neuroinflammation [45, 46], cerebrovascular integrity [47, 48], and susceptibility to infectious agents [49] should be considered. We also observed that the genes corresponding to the SNPs used for the PRS calculation in this study were involved in diverse pathogenesis of AD, such as cholesterol metabolism (*APOE, CLU, ABCA7*), tau toxicity (*BIN1, CD2AP, FERMT2, CASS4, PTK2B*), immune response (*CR1, CD33, MS4A, TREM2*), and endocytosis (*BIN1, PICALM, CD2AP, SPHA1, SORL1*) [3, 50].

Another finding in our study was that PRS_+*APOE*_ and PRS_−*APOE*_ had relatively lower predictability for the conversion of MCI to AD, compared to MRI and amyloid PET biomarkers, which implies a limitation of PRS use in disease stages when abnormalities on MRI and amyloid PET biomarkers have fully developed. However, PRS as a non-modifiable risk factor given at birth could offer early prediction, independent of the disease stage.

### Limitations and strengths

There are a few limitations in the current study. The sample size was small, so replication in an independent cohort would be desirable. With a longer follow-up period, some stable MCI participants might convert to AD. There are several challenges for PRS utility in clinical practice. Although the predictive utility of PRS has been validated in clinically well-defined and biomarker-confirmed cohorts, the PRS risk estimation in clinical populations from the community could be affected by variables such as ethnicity, environmental factors, and mixed pathologies. In addition, PRS as an additive indicator should be interpreted with caution to avoid ethical issues such as genetic determinism [51, 52]. Furthermore, bioinformatics tools for fast and simple data processing are needed for clinical PRS application in patients. With these issues being solved, PRS would be a useful biomarker based on the important genetic contributions in diseases and improve the cost and accessibility.

PRS is also an emerging tool for genetic risk estimation in AD. The predictability of PRS for disease progression has been reported with inconsistency depending on the inclusion or exclusion of *APOE* ε4 [4]. In this study, we not only investigated the predictability of PRS with and without *APOE* ε4, but also analyzed, for the first time, the interaction between PRS and *APOE* ε4. In addition, we present, for the first time, a comparison of the predictability of PRS for disease progression, with conventional imaging AD biomarkers including PET-based amyloid deposition and MRI-based brain atrophy.

## Conclusions

In summary, we demonstrated that PRS could predict conversion of MCI to dementia, showing a significant interaction between PRS and *APOE* ε4 carrier status, particularly stronger association in *APOE* ε4 non-carriers than in carriers. These results support that PRS can predict disease progression in MCI patients with no *APOE* ε4 allele, which has so far been the primary genetic risk factor used for clinical assessment. Furthermore, individualized risk estimation with PRS may allow timely interventions such as life style modification for disease prevention, which may reduce the dementia risk despite high genetic risk [53].

## Supplementary Information

**Additional file 1: Fig. S1.** The multidimensional scaling analysis for study population selection. **Table S1**. Number of SNPs for PRS calculation depending on GWAS association *P*-value thresholds. **Table S2**. Predictability of PRS for progression from mild cognitive impairment to dementia depending on GWAS *P*-value thresholds.

**Additional file 2: Table S3.** List of mapped genes.

## Data Availability

All demographic, imaging, and genetic data in this study are publicly available and can be downloaded from the ADNI database (adni.loni.usc.edu).

## Abbreviations

Aß: Beta-amyloid
AD: Alzheimer’s disease
ADNI: Alzheimer’s Disease Neuroimaging Initiative
BP: Biological processes
CC: Cellular components
CDR-SB: Clinical dementia rating sum of boxes
CI: Confidence interval
eQTL: Expression quantitative trait loci
GO: Gene ontology
GWAS: Genome-wide association studies
HR: Hazard ratio
LOAD: Late-onset Alzheimer’s disease
MAF: Minor allele frequency
MCI: Mild cognitive impairment
MF: Molecular functions
MMSE: Mini-Mental State Examination
PRS: Polygenic risk score
QC: Quality control
SNP: Single nucleotide polymorphism
SUVR: Standardized uptake value ratio
