# Supplementary material for: Predictability of polygenic risk score for progression to dementia and its interaction with APOE ε4 in mild cognitive impairment
Source: Transl Neurodegener. 2021 Aug 31;10:32. doi: 10.1186/s40035-021-00259-w (PMC8406896; doi:10.1186/s40035-021-00259-w)
Supplement: Supplementary file 1 — Additional file 1: Fig. S1. The multidimensional scaling analysis for study population selection. Table S1. Number of SNPs for PRS calculation depending on GWAS association P-value thresholds. Table S2. Predictability of PRS for progression from mild cognitive impairment to dementia depending on GWAS P-value thresholds. [file 40035_2021_259_MOESM1_ESM.docx]

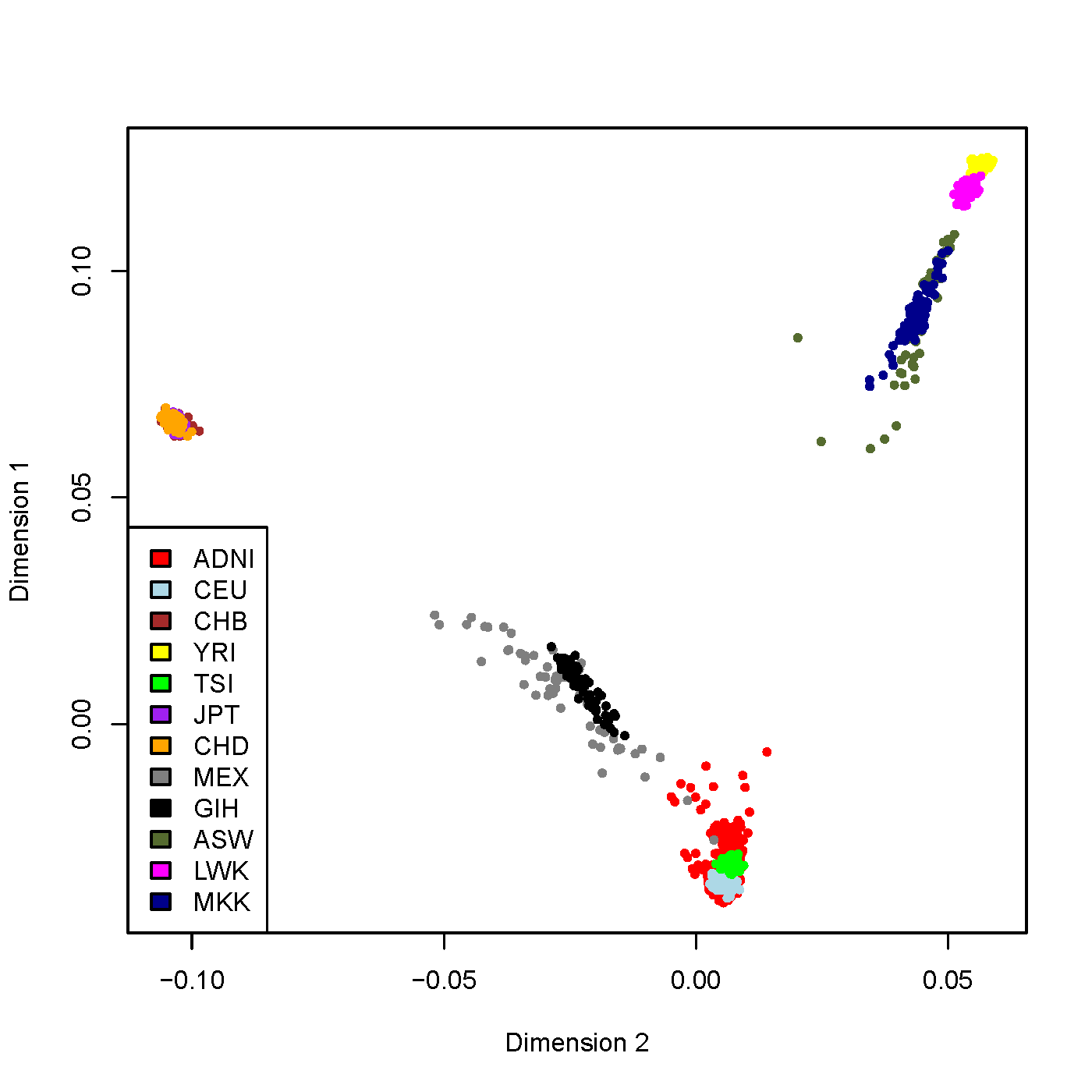
 **Fig S1. The multidimensional scaling analysis for study population selection.** Multidimensional scaling (MDS) plots of ADNI non-Hispanic White participants and genetic diversity across HapMap. ADNI non-Hispanic White participants were clustered to northern Europeans from Utah (CEU) and Tuscans from Italy (TSI) populations.

**Table S1. Number of SNPs for PRS calculation depending on the GWAS association *P*-value thresholds**

| GWAS association *P*-value threshold | PRS_+_*_APOE_* | PRS_-_*_APOE_* |
| --- | --- | --- |
| *P* < 5 × 10^-1^ | 558563 | 558045 |
| *P* < 5 × 10^-2^ | 93012 | 92753 |
| *P* < 1 × 10^-2^ | 23404 | 23214 |
| *P* < 1 × 10^-3^ | 3322 | 3199 |
| *P* < 1 × 10^-4^ | 608 | 513 |
| *P* < 1 × 10^-5^ | 204 | 122 |
| *P* < 1 × 10^-6^ | 125 | 57 |

PRS_+_*_APOE_*: PRS including SNPs within the 1Mb-region surrounding the *APOE* gene; PRS_-_*_APOE_*: PRS excluding SNPs within the 1Mb-region surrounding the *APOE* gene.

**Table S2. Predictability of PRS for progression from mild cognitive impairment to dementia depending on GWAS *P*-value thresholds**

|  | HR (95% CI), *P*-value | |
| --- | --- | --- |
|  | PRS_+_*_APOE_* | PRS_-_*_APOE_* |
| *P* < 5 × 10^-1^ | 1.085 (0.977–1.205), 1.27×10^-1^ | 1.054 (0.948–1.171), 3.31×10^-1^ |
| *P* < 5 × 10^-2^ | 1.222 (1.083–1.377), 1.06×10^-3^ | 1.149 (1.016–1.300), 2.65×10^-2^ |
| *P* < 1 × 10^-2^ | 1.251 (1.101–1.422), 5.68×10^-4^ | 1.116 (0.9454–1.167), 3.59×10^-1^ |
| *P* < 1 × 10^-3^ | 1.337 (1.215–1.559), 4.75×10^-7^ | 1.050 (0.945–1.167), 3.59×10^-1^ |
| *P* < 1 × 10^-4^ | 1.492 (1.347–1.652), 1.36×10^-14^ | 1.235 (1.081–1.410), 1.78×10^-3^ |
| *P* < 1 × 10^-5^ | 1.468 (1.335–1.615), 2.30×10^-15^ | 1.293 (1.157–1.445), 5.19×10^-6^ |
| *P* < 1 × 10^-6^ | 1.450 (1.319–1.593), 9.66×10^-15^ | 1.280 (1.163–1.408), 4.09×10^-7^ |

PRS_+_*_APOE_*: PRS including SNPs within the 1Mb-region surrounding the *APOE* gene; PRS_-_*_APOE_*: PRS excluding SNPs within the 1Mb-region surrounding the *APOE* gene.
